# Identification of the molecular determinants driving the substrate specificity of fungal lytic polysaccharide monooxygenases (LPMOs)

**DOI:** 10.1074/jbc.RA120.015545

**Published:** 2020-11-24

**Authors:** Kristian E.H. Frandsen, Mireille Haon, Sacha Grisel, Bernard Henrissat, Leila Lo Leggio, Jean-Guy Berrin

**Affiliations:** 1 INRAE, Aix-Marseille University, Polytech Marseille, UMR1163 BBF, Marseille, France; 2 Department of Chemistry, University of Copenhagen, Copenhagen, Denmark; 3 Architecture et Fonction des Macromolécules Biologiques (AFMB), CNRS, Aix-Marseille Université, Marseille, France; 4 INRAE, USC1408 Architecture et Fonction des Macromolécules Biologiques (AFMB), Marseille, France; 5 Department of Biological Sciences, King Abdulaziz University, Jeddah, Saudi Arabia

**Keywords:** copper monooxygenase, fungi, cellulose, plant cell wall, enzyme degradation, oligosaccharides, biorefinery, CBM, carbohydrate-binding module, Cell1, glucose, Cell2, cellobiose, Cell3, cellotriose, Cell4, cellotetraose, Cell5, cellopentaose, Cell6, cellohexaose, DP, degree of polymerization, GM, glucomannan, His-brace, Histidine brace, ICP-MS, inductively coupled plasma mass spectrometry, LC, C-terminal, LPMO, lytic polysaccharide monooxygenase, MLGs, mixed-linkage β-glucans, XG, xyloglucan

## Abstract

Understanding enzymatic breakdown of plant biomass is crucial to develop nature-inspired biotechnological processes. Lytic polysaccharide monooxygenases (LPMOs) are microbial enzymes secreted by fungal saprotrophs involved in carbon recycling. LPMOs modify biomass by oxidatively cleaving polysaccharides, thereby enhancing the efficiency of glycoside hydrolases. Fungal AA9 LPMOs are active on cellulose, but some members also display activity on hemicelluloses and/or oligosaccharides. Although the active site subsites are well defined for a few model LPMOs, the molecular determinants driving broad substrate specificity are still not easily predictable. Based on bioinformatic clustering and sequence alignments, we selected seven fungal AA9 LPMOs that differ in the amino-acid residues constituting their subsites. Investigation of their substrate specificities revealed that all these LPMOs are active on cellulose and cello-oligosaccharides, as well as plant cell wall–derived hemicellulosic polysaccharides, and carry out C4 oxidative cleavage. The product profiles from cello-oligosaccharide degradation suggest that the subtle differences in amino-acid sequence within the substrate-binding loop regions lead to different preferred binding modes. Our functional analyses allowed us to probe the molecular determinants of substrate binding within two AA9 LPMO subclusters. Many wood-degrading fungal species rich in AA9 genes have at least one AA9 enzyme with structural loop features that allow recognition of short β-(1,4)–linked glucan chains. Time-course monitoring of these AA9 LPMOs on cello-oligosaccharides also provides a useful model system for mechanistic studies of LPMO catalysis. These results are valuable for the understanding of LPMO contribution to wood decaying process in nature and for the development of sustainable biorefineries.

Efficient conversion of plant biomass for the production of biofuels and other sustainable bioproducts and materials is considered largely dependent on the key enzymes lytic polysaccharide monooxygenases (LPMOs) (1, 2). LPMOs are copper-dependent oxidoreductases that use molecular oxygen or hydrogen peroxide as cosubstrate to cleave polysaccharides (*e.g.* cellulose, hemicellulose, chitin, starch) through an oxidative mechanism (3, 4, 5). LPMOs exist either as single-domain enzymes or appended to other protein domains, for example, carbohydrate-binding modules (CBMs) (6, 7), directing the enzymes to their target substrate (8, 9, 10). To date, LPMOs are grouped into seven families in the CAZy database (11), namely the auxiliary activity (AA) families AA9-AA11 and AA13-AA16 (7), in which members have been found mainly in bacteria and filamentous fungi (12, 13, 14, 15, 16, 17), but also recently in arthropod and fern species (18, 19). In all LPMOs studied to date, the active-site redox center is made up of the strictly conserved histidine brace (His-brace) motif, which consists of the N-terminal His and a second His involved in the coordination of one copper atom (20, 21). The AA9 family also contains some members without any LPMO activity, lacking both the His-brace and copper, while features implicated in carbohydrate binding have been maintained (22).

Genes encoding AA9 LPMO enzymes are found in high numbers in fungal saprotrophs. For instance, species of the *Lentinus*, *Phanerochaete*, *Neurospora*, and *Podospora* genera display between 14 and 33 AA9 genes (https://mycocosm.jgi.doe.gov/mycocosm/home); however, the reason for this gene multiplicity is unknown. Structurally, these AA9 isoforms share a central β-sandwich core, but the surface-exposed structural features around the His-brace vary as reviewed (2, 21, 23). The LPMO structure from *Thermoascus aurantiacus* (*Ta*AA9A) (20) revealed a Cu-bound His-brace on a flat extended surface near a conserved Tyr, in the C-terminal (LC) loop, putatively involved in substrate binding. AA9 LPMOs were initially assumed to specifically target crystalline regions of polysaccharides, but later, the activity was also demonstrated on a range of hemicellulose polysaccharides such as xyloglucan (XG), xylan, glucomannan (GM), and mixed-linkage β-glucans (MLGs), and also on oligosaccharides (17, 24, 25, 26, 27, 28, 29, 30, 31). The AA9 LPMO from *Neurospora crassa* (*Nc*AA9C) is active on both cello-oligosaccharides and hemicellulosic substrates, suggesting that the enzyme recognizes small stretches of β-1,4-linked glucosyl units (24, 25). Borisova *et al.* (32) determined the structure of *Nc*AA9C and noticed three consecutive Asn residues in the L2 loop and an insertion in the L3 loop of *Nc*AA9C structure compared with other AA9 LPMOs (such as *Ta*AA9A) acting on insoluble cellulosic substrates (Fig. 1 and Fig. S1). Around the same time, we found that an AA9 LPMO from *Podospora anserina* (*Pa*AA9H) with an L3 loop similar to that of *Nc*AA9C showed activity on cello-oligosaccharides as well as XG and GM (29). Shortly after, we determined the structures of two AA9 LPMOs from *Lentinus similis* (*Ls*AA9A) and *Collariella virescens* (*Cv*AA9A, previously *Chaetomium virescens*), which both belong to the phylogenetic cluster C38 (see Results) and display activity on cello-oligosaccharides and on XG, MLG, and GM (28, 33). The L3 loops in *Ls*AA9A and *Cv*AA9A are slightly shorter than those in *Nc*AA9C and *Pa*AA9H; however, their L3 loops are all extended compared with those of *Ta*AA9A, which is not active on oligosaccharides. Interestingly, truncation of the L3 loop in *Nc*AA9C abolishes XG activity (34), and for a closely related (about 60% sequence identity) AA9 LPMO from *Chaetomium thermophilum* (denoted as *Ct*PMO1 [35]), a His-to-Ala mutation in the L3 loop (equivalent to His66 in *Ls*AA9A, see below) has been shown to abolish celloheptaose activity.

The first LPMO–carbohydrate complex structures determined were *Ls*AA9A in complex with cello-oligosaccharides (27, 36). Later, *Ls*AA9A was determined in complex with oligosaccharides derived from hemicelluloses (xylan, GM, and MLG) (28). The *Ls*AA9 complex structures confirmed that the oligosaccharides were bound by one Asn in the L2 loop, polar amino-acid residues in the L3 loop, and the conserved Tyr platform (Tyr 203) in the LC loop but also via charged residues in the L8 loop (after notation [37]). Subsites from −4 to +2 were defined based on the *Ls*AA9–cellohexaose (cell6) complex. The negative subsites (−4 to −1) were formed by the platform Tyr203 in the LC loop as well as Glu148, Asp150, and Arg159 in the L8 loop, whereas the positive subsites (+1 to +2) were formed by Asn28 in the L2 loop and His66 and Asn67 in the L3 loop. Interestingly, the structures also revealed an uncommon lone pair—π aromatic interaction between the pyranose ring O5 and the imidazole of the N-terminal His of the His brace (27, 36). Very recently, crystallographic cello-oligosaccharide complexes were also obtained with *Cv*AA9A and showed broadly the same interactions as *Ls*AA9A (Tandrup *et al*, submitted; 6YDC, 6YDD, and 6YDE) (33).

Although the substrate-binding determinants and active-site subsites are well defined for *Ls*AA9A, the molecular determinants of substrate specificity are still not easily predictable for other AA9 LPMOs. In this study, we focused on seven *Ls*AA9A-related enzymes from fungal saprotrophs to provide further insights into the main molecular determinants driving LPMO activity toward cello-oligosaccharides and hemicelluloses.

## Results

### Selection and production of seven AA9 LPMOs

The selection of AA9 sequences was carried out bioinformatically by searching for sequences with at least 50% sequence identity to *Ls*AA9A in the CAZy (http://www.cazy.org/) and JGI Mycocosm (https://mycocosm.jgi.doe.gov/mycocosm/home) databases. We retrieved 100 sequences mainly from basidiomycete species belonging to the Agaricomycetes class but also from ascomycete species belonging to the *Gyromitra* and *Aspergillus* genera (Pezizomycetes or Eurotiomycetes class, respectively). The vast majority of these species hold around 20 or more AA9 LPMO genes. Most of these fungi appeared only once in the list, whereas some appeared twice (*Gyromitra esculenta*, *Armillaria ostoyae*, *Crepidotus variabilis*, *Botryobasidium botryosum*, *Schizophyllum commune*, *Volvariella volvacea*) or even three or four times (*Crucibulum laeve* and *Coprinellus pellucidus*). It means that these fungi display at least one *Ls*AA9A-related LPMO that may have a dedicated biological function.

**Figure 1:**
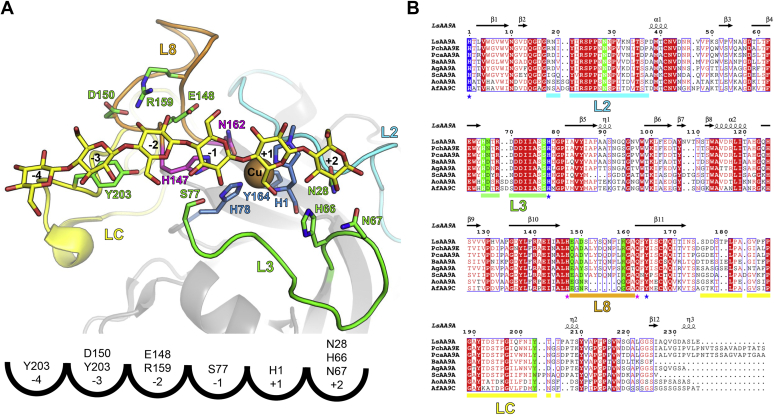
**Close-up view of *Ls*AA9A active site and sequence alignment of the AA9 LPMOs studied highlighting the residues putatively involved in substrate binding**. *A*, *Ls*AA9A-cell6 complex (PDB 5ACI) highlighting the subsites −4 to +2 and below schematic representation with residues contributing to substrate binding by hydrogen bonding or aromatic stacking or van der Waals interactions in each subsite. These residues are shown in sticks along with the residues involved in copper coordination (including the secondary sphere). The structural loop elements are colored as in panel *B*. *B*, multiple sequence alignment of the AA9 LPMOs investigated in this study. Positions involved in cello-oligosaccharide interactions and the His-brace motif are highlighted in *green* and *blue*, respectively. The positions of the primary and secondary coordination spheres of the copper are indicated with a *blue* or a *magenta star*, respectively. The L2, L3, L8, and LC loop regions are indicated with *cyan*, *green*, *orange*, and *yellow bars* below the sequences, respectively. LC, C-terminal; LPMOs, lytic polysaccharide monooxygenases.

*Ls*AA9A belongs to C38, and the 100 *Ls*AA9A-related sequences are classified within either subcluster C29 or C38, as previously defined by Lenfant *et al.* (6) based on the sequence conservation of AA9 N-terminal halves, that is, the N-terminal part of the sequences bordered by the residues forming the His brace. Of the 100 sequences, seven sequences were finally selected based on the composition of their putative substrate-interacting loops, L2, L3, L8, and LC (Fig. 1 and Fig. S1). The five AA9 sequences belonging to subcluster C38 originate from the basidiomycetes *Phanerochaete chrysosporium* (*Pch*AA9E JGI protein ID 2934397), *Phanerochaete carnosa* (*Pca*AA9A 261285), *Bjerkandera adusta* (*Ba*AA9A 353490), *Armillaria gallica* (*Ag*AA9A 500811), and *Schizophyllum commune* (*Sc*AA9A 2617723) and share between 63 and 76% sequence identity with *Ls*AA9A (Table 1). Two other AA9s belong to subcluster C29 and originate from the ascomycetes *Aspergillus oryzae* (*Ao*AA9A UniProt Q2US83) and *Aspergillus fumigatus* (*Af*AA9C GenBank EAL85444.1) (Fig. 1, Tables 1, and S1). *Ao*AA9A and *Af*AA9C naturally harbor a CBM1 module, and their catalytic AA9 domains share 53 and 55% sequence identity with *Ls*AA9A, respectively (Table 1). Some of the candidates chosen in this study originate from well-known fungal species for which other AA9 LPMOs have already been characterized, for example, *P. chrysosporium Pc*GH61D (38, 39) and *A. fumigatus Af*AA9B (40). However, no activity on oligosaccharides or hemicelluloses has been reported for any AA9 enzymes from these organisms.

**Table 1:** LsAA9A homologue sequences

Enzyme name	Organism	AA9 subcluster	Sequence identity to *Ls*AA9A	Substitutions	
				Negative subsites	Positive subsites
*Ls*AA9A	*Lentinus similis*	C38	-	n/a	n/a
*Pch*AA9E	*Phanerochaete chrysosporium*	C38	76%	E148Q	
*Pca*AA9A	*Phanerochaete carnosa*	C38	74%	E148Q	
*Ba*AA9A	*Bjerkandera adusta*	C38	69%	E148Q	N67D
*Ag*AA9A	*Armillaria gallica*	C38	64%	D150Y, R159K	N67D
*Sc*AA9A	*Schizophyllum commune*	C38	63%	Y203W	N67D
*Ao*AA9A	*Aspergillus oryzae*	C29	55%	D150N, S151R, R159K	N67D
*Af*AA9C	*Aspergillus fumigatus*	C29	53%	D150N, S151R, R159K	N67D

Subcluster 38 (C38), subcluster 29 (C29), negative subsites (−4, −3, −2, and −1), positive subsites (+1 and +2).

For each AA9 enzyme name, the organism of origin and the cluster they belong to are given. The sequences are listed by their identity to *Ls*AA9A and the substitutions affecting the subsite on either side of the scissile bond are indicated under *substitutions*.

Compared with *Ls*AA9A, the sequences of *Pch*AA9, *Pca*AA9, and *Ba*AA9 have a conservative substitution of the substrate-interacting Glu148 to a Gln near the −2 subsite, which we will refer to as E148Q. From here on, we will use this notation to refer to natural variations of the selected AA9 LPMOs compared with *Ls*AA9A (note that this notation does not refer to mutant variants). The sequence of *Ba*AA9 has an additional N67D substitution potentially affecting the +2 subsite. The sequences of *Ag*AA9, *Sc*AA9A, *Ao*AA9, and *Af*AA9 also have the N67D substitution together with other ones near the negative subsites. *Sc*AA9A has an Y203W substitution of the otherwise almost completely conserved platform Tyr involved in stacking interactions with the glycosyl unit in subsite −3. *Ag*AA9A has a D150Y substitution, and because the Asp in *Ls*AA9A interacts with the C6-hydroxyl of the glucosyl unit in subsite −3, it is possible that the Tyr in *Ag*AA9A is able to interact with C6 substitutions on β-(1,4)–linked glucans such as the xylose decorations found in XG. Thus, both Y203W and D150Y likely affect substrate binding in subsite −3 (Fig. 1). *Ao*AA9 and *Af*AA9 display some differences (D150N, S151R, or R159K) in the L8 loop, which is significantly shorter than the other six AA9s studied and similar to the equivalent loop in AA9 LPMOs acting on insoluble cellulose (such as *Ta*AA9A). Comparison of the *Ta*AA9A, *Ao*AA9A, and *Af*AA9C sequences indicate that an Arg residue is preserved at subsite −2, either at position 151 or 159.

To investigate the effect of natural variations at these functionally important positions, the catalytic domains of the seven selected AA9 LPMOs together with *Ls*AA9A (control enzyme from the subcluster C38) were produced in *Pichia pastoris*. After purification, inductively coupled plasma mass spectrometry analyses indicated that the proteins were correctly Cu-loaded, in agreement with the H_2_O_2_ production rates observed using the Amplex red assay (Table S1).

### The C29 and C38 AA9 LPMOs display activity on cellulose

The enzymatic activities of all *Ls*AA9A-related LPMOs were first assayed on two cellulosic substrates, amorphous cellulose (phosphoric acid swollen cellulose) and crystalline cellulose (Avicel), with cysteine as a reductant because our previous studies suggest that the use of cysteine eases the interpretation of the chromatograms compared with, for example, ascorbate (10, 29). As expected, we found that all these enzymes displayed some activity on cellulose (Table 2 and Fig. S2). After only 4-h reactions, we detected nonoxidized cello-oligosaccharides (with a degree of polymerization [DP] of up to 6) and peaks corresponding to C4-oxidized products (with retention times of about 25 min and 40 min [29]), but no evidence of C1 oxidation (cell2^ox^, cell3^ox^, and cell4^ox^). This clearly shows that all these AA9 LPMOs are C4-oxidizers when acting on substrates derived from cellulose. From overnight reactions with phosphoric acid swollen cellulose, we found that cellobiose (cell2), cellotriose (cell3), and cellotetraose (cell4) were the main products being formed, suggesting that these enzymes could be active on soluble cello-oligosaccharides (Fig. S2). The results obtained herein with *Ls*AA9A produced in *P. pastoris* are thus in line with previous reports on the substrate specificity and regioselectivity of the same enzyme recombinantly produced in *A. oryzae* (27, 28). This indicates that the lack of methylation of His1 in *P. pastoris* does not affect the product profile or the regioselectivity of the LPMO. Indeed, *Ls*AA9A is a strict C4-oxidizer on cellulose and releases a range of short cello-oligosaccharides dominated by cell2 and cell3, in contrast to the longer products such as cell4, cellopentaose (cell5), and cell6 released by *Ta*AA9A, which is not active on cello-oligosaccharides (27, 28).

**Table 2:** AA9 LPMO activities

Protein	Cellulose (PASC, Avicel)	XG	GM	MLG	Xylan	XXXGXXXG oligosaccharide	Cell6	Subsites	Cell5	Subsites	Cell4	Subsites	Cell3	Subsites
*Ls*AA9A	++	++	++	++	(+)	++	++	−4 to +2	++	−3 to +2	+	−2 to +2	+	−1 to +2
*Pch*AA9E	++	++	++	+	-	++	++	−4 to +2	++	−3 to +2	+	−2 to +2	-	-
*Pca*AA9A	++	++	++	+	-	++	++	−4 to +2	++	−3 to +2	+	−2 to +2	-	-
*Ba*AA9A	++	-	-	-	-	+	++	−4 to +2	ND	-	-	-	ND	-
AgAA9A	++	++	+	+	-	++	++	−4 to +2	ND	-	(+)	(−2 to +2) (−3 to +1)	ND	-
*Sc*AA9A	++	-	-	(+)	-	+	++	−3 to +3	ND	-	-	-	ND	-
*Ao*AA9A	++	++	+	+	-	++	++	−4 to +2	++	−3 to +2	+	−2 to +2	ND	-
*Af*AA9C	++	++	+	+	-	++	++	−4 to +2	++	−3 to +2	+	−2 to +2	ND	-

XG, xyloglucan; GM, glucomannan; MLG, mixed-linked β-glucans; LPMO, lytic polysaccharide monooxygenase; cell4, cellotetraose; cell5, cellopentaose; cell6, cellohexaose; ND, not determined.

*Ls*AA9A activities on cell5 and cell3 are inferred from literature (27, 28).

For each AA9 enzyme relative semiquantitative activities for one given substrate are indicated with “++”, “+”, “(+)”, or “-” for high activity, intermediate activity, little activity, or no activity, respectively. Subsites indicate which subsites the substrate occupy.

### The C29 and C38 AA9 LPMOs display activity on cello-oligosaccharides

We assayed the activity of the selected AA9 LPMOs on cello-oligosaccharides (cell3-cell6) in the presence of cysteine. All AA9 LPMOs tested were active on cell6 (Table 2), generating both cell3 and cell4 as the main nonoxidized products (Table 3 and Fig. 3), with constant rates over a 2-h reaction. We clearly found that no C1-oxidized products were formed and the chromatograms did indicate peaks corresponding to C4-oxidized species eluting around 25 and 40 min. Although the latter were not easily observable because of low sensitivity of species with late elution times, it strongly suggests that these AA9 LPMOs display a C4 regioselectivity in agreement with previous data on *Ls*AA9A.

**Table 3:** Product profiles and initial rates of AA9 LPMO reactions with cello-oligosaccharides

Protein	Molar ratios after 2-h reaction with cell6 (%)			Change in Cell6 peak area (Δarea/min)	Fit of linear regression (R^2^)	Rate of cell6 conversion (μM ×min^−1^)	Rate of Cell6 conversion pr. enzyme (min^−1^)	Change in Cell5 peak area (Δarea/min)	Fit of linear regression (R^2^)	Rate of cell5 conversion (μM ×min^−1^)	Rate of Cell6 conversion pr. enzyme (min^−1^)
	Cell6	Cell4	Cell3								
*Ls*AA9A	59	24	17	ND	ND	ND	ND	ND	-	-	-
*Pch*AA9E	68	23	9	−0.12	0.94	3.0	0.8	−0.20	0.99	5.0	1.3
*Pca*AA9A	49	32	20	−0.14	0.98	3.5	0.9	−0.18	0.99	4.5	1.1
*Ba*AA9A	83	11	6	ND	ND	ND	ND	ND	-	-	-
*Ag*AA9A	68	20	12	−0.10	0.98	2.5	0.6	ND	-	-	-
*Sc*AA9A	62	12	25	−0.13	0.97	3.3	0.8	ND	-	-	-
*Ao*AA9A	57	31	12	−0.13	0.93	3.3	0.8	−0.18 ± 0.05	0.95 ± 0.03	4.5	1.1
*Af*AA9C	55	33	12	−0.15	0.95	3.8	0.9	−0.22 ± 0.02	0.97 ± 0.02	5.5	1.4

cell6, cellohexaose; cell5, cellopentaose; cell4, cellotetraose; LPMO, lytic polysaccharide monooxygenase; ND, not determined.

Areas of peaks corresponding to nonoxidized products and substrate (cell3, cell4 and cell6) after 2-h reaction were converted to molar ratios using the following relationship; area = 4 × [cello-oligosaccharide]. The change in the substrate peak area (Δ cell5 or Δ cell6) were monitored using six time points; 0 min, 20 min, 40 min, 60 min, 90 min, 120 min, and linear regression was calculated based on these data points. The numerical value of the slopes was converted to molar rates, and the 100× dilution factor was applied to obtain the final rates of conversion of cell5 and cell6. The rate of cell6/cell5 conversion pr. enzyme is calculated by dividing the rate with the enzyme concentration (4 μM). Reactions with cell5 and *Ao*AA9A or *Af*AA9C were run in triplicates. Rates were not calculated for *Ls*AA9A and *Ba*AA9A because the data points did not fit the regression properly.

**Figure 2:**
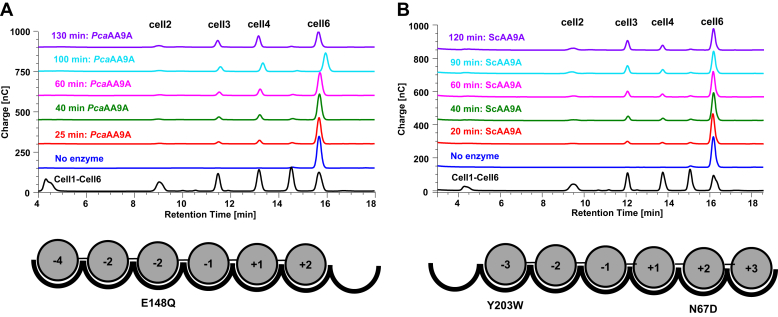
**Time-course reaction of cellohexaose degradation**. Comparison of the preferred binding mode between the following: *A*, *Phanerochaete carnosa* AA9 (*Pca*AA9A) and *B*, *Schizophyllum commune* (*Sc*AA9A). Schematic representations below the chromatograms indicate amino-acid substitution compared with *Ls*AA9A and subsite organization.

The majority of the AA9s tested produced cell4 as the major nonoxidized product followed by cell3, except for *Sc*AA9A, which displayed a different profile because cell3 was the major product (Fig. 2, Tables 2 and 3). This interesting observation indicates that the majority of the AA9 enzymes can bind cell6 preferably from subsite −4 to +2 in agreement with previous biochemical and structural reporting on *Ls*AA9A (*e.g.* in supplementary figures 1 and 4 of [27]). However, based on our observations, we believe that the preferred binding mode of *Sc*AA9A is from subsite −3 to +3. The rates of substrate conversion (and product formation although not shown) were determined and under our experimental conditions, we found that the majority of AA9 LPMOs converted cell6 with relatively similar constant rates of around 1 min^−1^ (Table 3).

**Figure 3:**
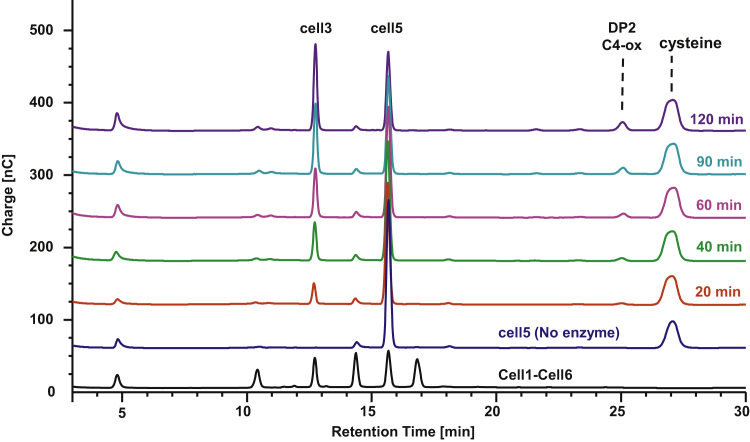
**Time-course reaction of cellopentaose conversion exemplified by *Pch*AA9E**. Reactions with cellopentaose (cell5) analyzed after 20, 40, 60, 90, and 120 min. Cleavage of cell5 by *Pch*AA9E produced cell3 (ca. 13-min retention time) and C4-oxidized cellobiose (ca. 25 min retention time) indicating that these enzymes are C4 oxidizers binding cell5 in a single binding mode. cell3, cellotriose.

When investigating LPMO activity on cello-oligosaccharides smaller than cell6 (<DP6), we found, just like for *Ls*AA9A, that *Pch*AA9E, *Pca*AA9A, *Ao*AA9A, and *Af*AA9C were clearly able to degrade cell5 and cell4, whereas weak activity was observed for *Ag*AA9A. *Pch*AA9E and *Pca*AA9A, the AA9 LPMOs most closesy related to *Ls*AA9A, showed no activity with cell3 as substrate unlike *Ls*AA9A (27). With cell5 as substrate, we found that *Pch*AA9E, *Pca*AA9A, *Ao*AA9A, and *Af*AA9C (exemplified by *Pch*AA9E, Fig. 3) generated cell3 as the major nonoxidized product and C4-oxidized DP2 products (and also a minor peak corresponding to glucose emerged during 2-h reaction). This indicates that the preferred binding mode of cell5 is from subsites −3 to +2 similarly to *Ls*AA9A. We monitored the conversion of cell5 and the corresponding formation of cell3, which occurred at a constant rate over a 2-h time course (Table 3 and Fig. 3). Again, we found that the four enzymes appeared to convert the cell5 substrate at similar rates of around 1 min^−1^ (Table 3). The small formation of glucose could arise from some scarce cell3 degradation that occured under these experimental conditions, although we did not observe activity when running experiments with only cell3 as a substrate.

With a cell4 substrate, we found that the four enzymes produced mainly cell2, confirming the binding mode from the −2 to +2 subsites, as previously reported for *Ls*AA9A (27, 28). In contrast, *Ag*AA9A appeared to equally produce both cell2 and glucose, although the activity was low, indicating binding from both −2 to +2 and −3 to +1 (Table 2). Incubating any of the substrates in the absence of enzyme or replacing the enzyme with equimolar Cu-acetate resulted in no substrate conversion or product formation.

### The C29 and C38 AA9 LPMOs display activity on hemicellulose-derived substrates

**Figure 4:**
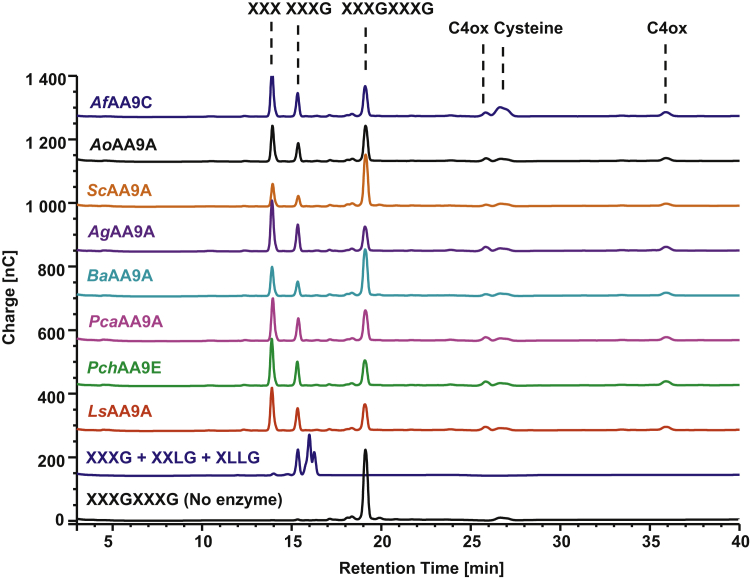
**Activity of AA9 LPMOs on xyloglucan**. Cleavage of XXXGXXXG by the AA9 LPMOs after a 24-h reaction. The major nonoxidized product is XXX, but also XXXG is formed indicating that an unsubstituted glucosyl unit (G) is preferred in subsite +1 and that a C6-xylose substituted glucosyl unit (X) is tolerated. The XG-oligosaccharide standards were previously found to elute in following order—XXXG, XXLG, and XLLG (24, 30). LPMOs, lytic polysaccharide monooxygenases; XG, xyloglucan.

**Figure 5:**
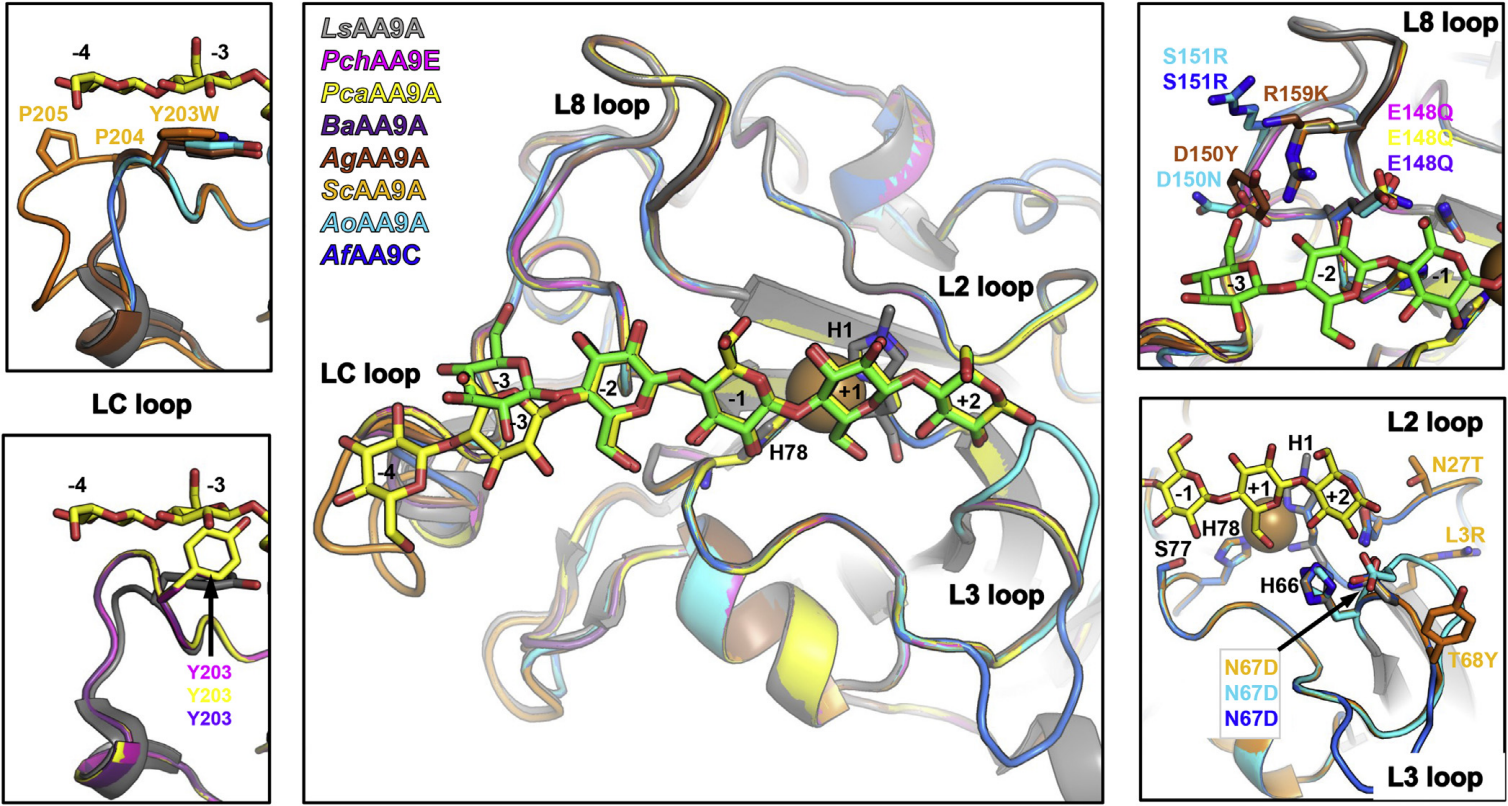
**Homology models of AA9 LPMOs**. Loop elements of each model are colored as follows—*Pch*AA9E (*magenta*), *Pca*AA9A (*yellow*), *Ba*AA9A (*purple*), *Ag*AA9A (*brown*), *Sc*AA9A (*orange*), *Ao*AA9A (*cyan*), and *Af*AA9C (*blue*). *Ls*AA9A structures are shown transparent in *gray*. All β-strands are in *gray*, and the L2, L3, L8, and LC loops are indicated with *black* labels. The insets highlight differences of the models in loops L2 and L3 (*lower right panel*), L8 (*upper right panel*), and LC (*left panels*). Labels are colored with according to the color of the model. *Black* labels apply to *Ls*AA9A. Cellopentaose (from the *Ls*AA9A-cell5 complex PDB 5NLS) and cellohexaose (from the *Ls*AA9A-cell6 complex PDB 5dsACI) are shown in *green* and *yellow*, respectively. LC, C-terminal; LPMOs, lytic polysaccharide monooxygenases.

Next, we tested the ability of the enzymes to degrade an XG polysaccharide from tamarind seeds. The reaction products were analyzed with chromatography using XXXG, XXLG, and XLLG oligosaccharides as standards (nomenclature after (41) where G and X denote β-(1,4)–linked glucosyl units either nonsubstituted or with xylosyl substitutions at the C6 position, respectively). For the majority of AA9s, we observed significant activity except for *Ba*AA9A and *Sc*AA9A where activity could not be detected even after overnight incubation (Fig. S4*A*). Among the products from 1-h reactions (Fig. S4*B*), three peaks with retention times around 15 min could correspond to short nonoxidized oligosaccharides (such as XXXG) and peaks with retention times 20 to 25 min, which could correspond to longer native or C4-oxidized oligosaccharides (after comparison with [24]). After overnight reactions (ca. 22 h, Fig. S4*A*), these peaks were significantly smaller, indicating a possible activity of these enzymes on XG oligosaccharides. Therefore, we tested the ability of these AA9 LPMOs to degrade an XG XXXGXXXG tetradecasaccharide. Reactions with XXXGXXXG showed that all AA9 (including *Ba*AA9A and *Sc*AA9A) cleaved XXXGXXXG, which lead to a small peak corresponding to XXXG and a larger peak corresponding likely to an XXX hexasaccharide (Fig. 4). This suggests that these AA9 LPMOs preferentially bind the XXXGXXXG nonreducing end in subsite −3 and with a nonsubstituted glucosyl unit in the +1 subsite, which would generate XXX and C4-oxidized GXXXG species, as also reported by Agger *et al.* (24). They can also accommodate a xylose-substituted glucosyl unit in subsite +1 and bind the XXXGXXXG nonreducing end in subsite −4 producing a nonoxidized and a C4-oxidized XXXG species. We also speculated that the D150Y substitution near subsite −3 in *Ag*AA9A (Fig. 5) could enhance binding of a C6-substituted glucosyl unit, and thus, *Ag*AA9A would have the strongest preference for binding XXXGXXXG. Reactions with XXXGXXXG and *Ag*AA9A or *Ls*AA9A were run in parallel for 20 min, 2 h, and 24 h (Fig S5); however, the results of the time-course reaction with XXXGXXXG did not reveal any striking difference between *Ag*AA9A and *Ls*AA9A.

Finally, we tested the ability of the AA9 enzymes to cleave other hemicellulose substrates such as MLG, GM, and xylan. For many of the AA9s, products following enzymatic cleavage were detectable from reactions with MLG and GM. However, for *Sc*AA9A, we only found weak activity on MLG and for *Ba*AA9A, no clear activity on either of MLG or GM (Table 2). No clear activity on xylan or xylohexaose was observed for any of the *Ls*AA9A-related LPMOs.

### Homology models of the studied LPMOs

To better interpret and discuss the biochemical results, homology modeling was carried out using *Ls*AA9A as the template. All models were highly reliable and with reasonable geometric quality (Table S2) according to Swiss-Model scores (42). Although still reliable, the *Ao*AA9A model had the lowest quality as expected, given its lowest sequence identity (∼55 %) to the template (Table 1 and Fig. S6*A*). The LC loops bearing the Tyr/Trp residue expected to interact with the substrate (see Discussion) are the regions of the homology models that are structurally most different from the *Ls*AA9A template. However, some caution must be applied in interpretation because these regions are also less reliably modeled (Fig S6).

## Discussion

Here, we have investigated substrate specificities of seven fungal AA9 LPMOs from two related phylogenetic subclusters (C29 and C38) and shown activity on both cello-oligosaccharide- and hemicellulose-derived substrates. Our results support the notion that activity on short cello-oligosaccharides correlate with the activity on plant cell wall β-(1,4)–linked hexose chains. All of the LPMOs behaved relatively similarly to the canonical *Ls*AA9A in terms of regioselectivity, ability to degrade cello-oligosaccharides and hemicelluloses containing β-(1,4)–linked D-glucose in contrast to AA9 from the other groups, which are mostly only active on cellulose. Our results show that all of the investigated LPMOs could bind cell6 from both subsite −4 to +2 and −3 to +3, indicating that the LPMOs in fact recognize five β-(1,4)–linked glucosyl units as in cell5. Indeed, we found a single binding mode for cell5 (from subsites −3 to +2), and both cell5 and cell6 are degraded with similar rates (indicating that subsites −4 and +3 are not strong binding sites). The very similar and low rates measured could be an indication of H_2_O_2_ being a limiting factor. It would be interesting to further investigate the activity of these LPMOs using H_2_O_2_ as cosubstrate under more controlled conditions. Based on the set of AA9 LPMOs investigated in this study, we conclude that in general, an extended L3 and an L8 loop both with polar/charged amino acid residues are determinants of cello-oligosaccharide activity in AA9 LPMOs. These features are present in AA9 LPMOs from C29 and C38 and small sequence differences within the L3, L8, and LC loops do not significantly impact the activity.

Although the AA9 LPMOs investigated here are overall similar, in-depth biochemical analyses revealed some subtle differences between the enzymes studied in terms of activity and substrate binding. For instance, *Ls*AA9A is able to cleave cell3 (bound by Glu148 and Asn67 in subsites −1 to +2, respectively), unlike the other AA9 LPMOs investigated here. We found that *Pch*AA9E and *Pca*AA9A (most closely related to *Ls*AA9A) cleave cell4 as the smallest oligosaccharide substrate (from subsites −2 to +2). Structural modeling shows that Gln148 of *Pch*AA9E and *Pca*AA9A align well with the *Ls*AA9A Glu148. It is possible that E148Q alter the H-bond, which weakens cell3 binding, which could explain the absence of cell3 activity (Fig. 5). It also appears that N67D and R159K lead to loss of cell4 activity unless compensated by other substitutions in the L8 loop such as D150N and S151R found in *Ao*AA9A and *Af*AA9C (Fig. 5 and Fig. S6*A*). Our results also suggest that the double substitution N67D/E148Q negatively affects cello-oligosaccharide binding because the *Ba*AA9A enzyme only showed activity on cell6 and not on cello-oligosaccharides < DP6 (Table 2).

The *Sc*AA9A enzyme displayed a different product profile by binding cell6 from −3 to +3. A weakened −4 subsite or a strengthened +3 subsite could explain this observation. Indeed, *Sc*AA9A displays a Trp in the LC loop instead of the otherwise conserved substrate-interacting platform Tyr. Based on the *Sc*AA9A model, these aromatic side chains likely make a similar interaction with cello-oligosaccharides, although two Pro residues (Pro204 and Pro205, not found in the other investigated enzymes) appear to affect the conformation of the LC loop (Fig. 1*B*, Fig. 5, and Fig. S6*C*). We believe that these subtle differences could alter the accommodation of the substrates in the −4 subsite and that the Trp preferably interacts with the terminal glucosyl unit at the nonreducing end in subsite −3. The *Sc*AA9A 3D model also indicate that the T68Y and N27T substitutions (in loops L3 and L2, respectively), which are the neighboring residues of those making up subsite +2, and the L3R could play a role in binding of the terminal glucosyl unit at the reducing end in subsite +3 (Fig. 5, bottom right inset). Both of these explanations would lead to preferential binding of cell6 from −3 to +3.

A recently determined structure of *Cv*AA9A (which shares 45% sequence identity with *Ls*AA9A and also belongs to subcluster C38) with cell6 bound between subsite −3 and +3 (PDB code 6YDE) shows that the *Cv*AA9A Arg3 is involved in a water-mediated hydrogen-bonding network in contact with the glucosyl unit at subsite +3 (33). In addition, both biochemical and structural analyses show that *Cv*AA9A binds cell4 from −3 to +1 (PDB code 6YDC) unlike the AA9 LPMOs investigated here.

We also found some differences when investigating the conversion of hemicellulose substrates. In the *Ag*AA9A 3D model, a Tyr (D150Y) is positioned next to the Tyr platform near subsite −3 (Fig. 5 and Fig. S6*B*) and could play a role in XG binding through interaction with xylose substitutions at the C6-hydroxyl of the glucosyl unit. However, the XG activity of *Ag*AA9A was not markedly higher than that of other AA9 LPMOs characterized here (Fig. S4). Somewhat puzzling, however, we found that *Ba*AA9 and *Sc*AA9A cleaved an XXXGXXXG oligosaccharide but did not show any activity on the XG polysaccharide substrate, unlike the rest of the characterized AA9 LPMOs. Finally, although *Ls*AA9A is able to cleave xylan and xylohexaose (albeit much less efficient than cellulose substrates), we did not find any significant activity on these substrates with any of the other C29 or C38 AA9s investigated here. Thus, *Ls*AA9A remains the only AA9 LPMO with reported activity on isolated xylan (as found here and previously [28]).The AA9 LPMOs from subclusters C29 and C38 (modular AA9-CBM1 proteins or single-domain AA9 enzymes, respectively) characterized in this study all originate from fungal species commonly associated with saprotrophic and/or plant parasitic lifestyles. Many of these are *Agaricomycetes* fungal species that display multiple AA9 genes, but on average only one or two putative cello-oligosaccharide–active AA9 LPMOs. During this study, we have also noted that some AA9 sequences, grouped in the neighboring subcluster C27, have similar putative cello-oligosaccharide binding features and are appended to a module of unknown function. These sequences originate mostly from plant pathogen species of the *Dothideomycetes* class, and it would be interesting to see if there is a correlation between cello-oligosaccharide specificity and pathogenicity.

We and others have previously observed a correlation between LPMO activity on hemicelluloses containing β-(1,4)–linked D-glucose (24, 25, 29, 35) and cello-oligosaccharides for some AA9s (*e.g. Nc*AA9C, *Pa*AA9H, and *Ct*PMO1) from other subclusters (C21 and C23, which contain many *Sordariomycetes* species and are not closely related to C38 or C29) (Fig. S1). These AA9 LPMOs behave similarly to the AA9 LPMOs investigated in this study. This shows that the LPMO activity on both cello-oligosaccharides and hemicelluloses is not limited to AA9s in a few closely related phylogenetic subclusters but is found more widely in a number of fungal phylogenetic classes in species with different lifestyles. Interestingly, a recent transcriptomic study on *P. carnosa* showed that the gene encoding *Pca*AA9A (studied herein) was expressed during several days on growth on white spruce (*Picea glauca*) and aspen (*Populus tremuloides*) (43). In another study on *Aspergillus nidulans*, the gene encoding AN1602 (44) (an AA9-CBM1 protein displaying 65% sequence identity with *Ao*AA9 and *Af*AA9) was highly transcribed during growth on cellulose and xylan, and the enzyme was shown to be active on both cell6 and XG (44, 45). Recently, XG and cell6 activities have also been reported for an AA9-CBM1 LPMO from *Aspergillus tamarii* (*At*AA9A) for which the catalytic AA9 domain shares about 80% sequence identity with *Ao*AA9A and *Af*AA9C (46).

It seems that many wood-decaying fungal species use an arsenal of AA9 LPMOs with at least one AA9 member that is able to recognize a short β-(1,4)–linked glucan motif conferring activity on cello-oligosaccharides and hemicellulosic substrates. The functional features of the fungal AA9 LPMOs studied herein do not fit with the common belief that LPMOs are specifically targeting crystalline regions of cellulose fibrils. Rather, C29 and C38 LPMOs seem to target a variety of complex motifs within the plant cell wall. Whatever is their biological function, we have shown that is it possible, from their sequence, to predict their LPMO activity toward cello-oligosaccharides and hemicellulose substrates, although their precise substrate specificity remains difficult to predict. In addition, the AA9 LPMOs displaying a single oligosaccharide-binding mode could be useful model systems for further mechanistic investigations.

## Experimental procedures

### Cloning and expression of AA9 LPMOs

All proteins were produced using the in-house 3PE Platform (*Pichia Pastoris* Protein Express; www.platform3pe.com/) (47). Genes encoding *Ls*AA9A (GenBank ALN96977.1), *Af*AA9C (EAL85444.1), *Ao*AA9A (UniProt Q2US83), *Pch*AA9E (JGI protein ID 2934397), *Pca*AA9A (261285), *Ba*AA9A (353490), *Ag*AA9A (500811), and *Sc*AA9A (2617723) were synthetized after codon optimization for *P. pastoris* (GeneScript, Piscataway, NJ) and inserted into the vector pPICZαA (Invitrogen, Cergy-Pontoise, France) using BstBI and XbaI restriction sites in frame with the C-terminal (His)_6_-tag.

*P. pastoris* strain X33 and the pPICZαA vector are components of the *P. pastoris* Easy Select Expression System (Invitrogen). The resulting pPICZαA plasmid containing the *AA9* gene was linearized with PmeI, and competent *P. pastoris* X33 was transformed using electroporation as described elsewhere ([29, 47] and reference herein). Zeocin-resistant *P. pastoris* transformants were then screened for protein production (47). Protein candidates suitable for upscaled production were produced in 2-L flasks in which cells were grown in 500-ml (buffered complex glycerol medium containing 1 ml L^−1^ Pichia trace elements (PTM_4_) salts and buffered with 100-mM phosphate, pH 6.0) at 30 °C in an orbital shaker (200 rpm) for 16 h until an absorbance of ∼ 2 to 6 at 600 nm. The cells were pelleted by centrifugation (10 min at 2700*g*) at room temperature and resuspended in 100 ml of (buffered complex methanol medium) containing 1 ml L^−1^ (PTM_4_) salts at 20 °C in an orbital shaker (200 rpm) for 3 days with daily addition of 3% methanol (v/v). The culture supernatants were collected by centrifugation at 2700*g* for 10 min at 4 °C. The pH was adjusted to 7.8 just before purification and filtered using 0.22-μm filters (Millipore, Molsheim, France).

### Purification and copper loading of AA9 LPMOs

Purification was performed using an AKTAxpress system (GE healthcare). The supernatants were loaded onto 5-ml His Trap HP columns (GE healthcare, Buc, France) equilibrated in 50-mM Tris-HCl, pH 7.8, 150-mM NaCl, and 10-mM imidazole (buffer A), and sample loading was finished by an additional two column volumes of buffer A. The proteins were eluted with imidazole in a stepwise manner with 2% (10 mM), 50% (250 mM), and 100% (500 mM) of buffer B (50-mM Tris HCl, pH 7.8, 150-mM NaCl, 500-mM imidazole). Fractions containing recombinant enzymes were pooled and concentrated with a 10-kD vivaspin concentrator (Sartorius, Palaiseau, France) to remove imidazole and buffer exchanged in 50-mM 2-(N-morpholino)ethanesulfonic acid buffer, pH 6.0. The concentration of purified proteins was determined by absorption at 280 nm using a Nanodrop ND-2000 spectrophotometer (Thermo Fisher Scientific), with calculated molecular mass and molar extinction coefficients derived from the sequences. Proteins were loaded onto 10% SDS-PAGE gels (Thermo Fisher Scientific, IL), which were stained with Coomassie Blue. The molecular mass under denaturating conditions was determined with reference standard proteins (PageRuler Prestained Protein Ladder, Thermo Fisher Scientific). The concentrated proteins were incubated with Cu-acetate (slight in excess) for either 1 h at room temperature or overnight at 4 °C. Excess of copper was removed by loading onto a HiLoad 16/600 Superdex 75 Prep Grade column (GE healthcare) and separated in 20-mM Na-acetate, pH 5.5, and 100-mM NaCl. The copper-loaded and purified batches (prepared in 20 mM Na-acetate pH 5.5 and 100 mM NaCl) were used for the Amplex Red Assay (48, 49), inductively coupled plasma mass spectrometry analysis (as previously described [13]), and enzymatic reactions.

### AA9 LPMO enzymatic reactions

Reactions with AA9 enzymes (at 4-μM concentration) were carried out at 50 °C at 850 rpm in 20-mM Na-acetate, pH 5.5, 100-mM NaCl in the presence of 1 mM cysteine, chosen in preference to other reducing agents for easier interpretation of the chromatograms. For polysaccharides, incubation times were either 1 h or overnight (approximately 20 h) and reaction mixtures were diluted ten times before injection. XG from tamarind seeds (CAS No. 37294-28-3) was purchased from Megazyme, and XXXGXXXG was kindly provided by H. Brumer (UBC, Vancouver, Canada). It was prepared as described in (50). For cello-oligosaccharides (initial concentration of 1 mM), time-course reactions were carried out with time points at 20, 40, 60, 90, and 120 min, and the reaction mixtures injected to the system were x100 dilutions. For the C29 AA9s, the 2 h time-course reactions with cell5 were run in triplicates. For time-course experiments with cell5, the peak areas of the different reaction time points were plotted against time and the slope was converted to molar rates. Control reactions with LPMO and a cello-oligosaccharide in the absence of an electron donor showed no product formation. For all substrates, negative controls were run both without enzyme and in the presence of Cu-acetate in equimolar concentrations to the enzymes (*i.e.* 4 μM). None of the negative controls with Cu-acetate produced peaks above the background.

### Analysis of carbohydrate products

Analysis of carbohydrate products (both oxidized and nonoxidized) after incubation of polysaccharide and oligosaccharide substrates with AA9 LPMOs was performed using high-performance anion-exchange chromatography coupled with pulsed amperometric detection as described in (51). By the use of standards (Megazyme), peak areas were converted to molar concentration. A peak corresponding to the electron donating redox partner cysteine was observed with retention time of 26 to 27 min. Peaks at retention times corresponding to oxidized products have been previously reported (29).

### Homology modeling

Swiss-Model (https://swissmodel.expasy.org/) (42) was chosen because we found this method to better handle the methylated His and the position of the Tyr platform (Tyr203 in *Ls*AA9A) than other available methods (*e.g*. the Phyre2 server (http://www.sbg.bio.ic.ac.uk/) [52]). Swiss-Model runs through the server at https://swissmodel.expasy.org/ with template PDB 5ACH (the closest available for all investigated enzymes) (42). GQME and QMEAN Z-scores used to assess model quality are summarized in (Table S2). The lowest scores in terms of reliability and geometry were GQME of 0.77 and QMEAN Z-score of −2.09, which are still very reasonable for a useful model. MolProbity overall scores are similar for models and template, with models generally having slightly worse Ramachandran statistics.

## Data availability

All data generated or analyzed during this study are contained within this article.

## Conflict of interest

The authors declare that they have no conflicts of interest with the contents of this article.
