# Supplementary material for: Identification of the molecular determinants driving the substrate specificity of fungal lytic polysaccharide monooxygenases (LPMOs)
Source: J Biol Chem. 2020 Nov 24;296:100086. doi: 10.1074/jbc.RA120.015545 (PMC7949027; doi:10.1074/jbc.RA120.015545)
Supplement: Supplementary file 1 — Supplementary Tables and Figures [file mmc1.pdf]

Investigation of seven fungal lytic polysaccharide monooxygenases (LPMOs) underpins key molecular determinants driving broad substrate specificity

Kristian E. H. Frandsen[1,5], Mireille Haon[1], Sacha Grisel[1], Bernard Henrissat[2,3,4], Leila Lo Leggio[5], Jean-Guy Berrin[1,*]

Supplementary Table 1

Supplementary Table 2

Supplementary Figure 1

Supplementary Figure 2

Supplementary Figure 3

Supplementary Figure 4

Supplementary Figure 5

Supplementary Figure 6

Table S1

Table S1. *Ls*AA9A homologues sequences								
**Protein**	**Phylum**	**Class**	**Order**	**Organism**	**Seq. identity to *Ls*AA9A**	**Production Yields***	**Cu:Protein ratio (ICP-MS)**	**AR assay mean slope**
*Ls*AA9A	Basidio	Agaricomycetes	Polyporales	*Lentinus similis*	-	110	0.4	387 ± 9
*Pch*AA9E	Basidio	Agaricomycetes	Polyporales	*Phanerochaete chrysosporium*	76 %	20	1.3	434 ± 81
*Pca*AA9A	Basidio	Agaricomycetes	Polyporales	*Phanerochaete carnosa*	74 %	10	1.1	427 ± 6
*Ba*AA9A	Basidio	Agaricomycetes	Polyporales	*Bjerkandera adusta*	69 %	5	1.6	466 ± 25
*Ag*AA9A	Basidio	Agaricomycetes	Agaricales	*Armillaria gallica*	64 %	65	0.9	345 ± 6
*Sc*AA9A	Basidio	Agaricomycetes	Agaricales	*Schizophyllum commune*	63 %	25	0.7	347 ± 5
*Ao*AA9A	Asco	Eurotiomycetes	Eurotiales	*Aspergillus oryzae*	55 %	40	1.1	434 ± 30
*Af*AA9C	Asco	Eurotiomycetes	Eurotiales	*Aspergillus fumigatus*	53 %	80	1.1	348 ± 7

Basidio (Basidiomycetes)
Asco (Ascomycetes)
MeOH (methanol)
*mg/L methanol media
Amplex Red (AR) assay (ΔAbs_{595} / min)

Table S2

**Table S2. Homology models statistics**					
	**Sequence identity to template (blastp/SwissModel)**	**GMQE***	**QMEAN Z-score#**	**Molprobity overall score (SwissModel implementation)**	**Ramachandran favoured**
Template 5ACH	-	-	-	2.03	92.67%
*Pch*AA9E	75%/77%	0.83	-0.29	1.70	88.44%
*Pca*AA9A	73%/75%	0.81	-0.90	1.72	88.00%
*Ba*AA9A	68%/70%	0.83	-0.76	1.90	90.19%
*Ag*AA9A	62%/66%	0.85	-1.30	1.82	91.20%
*Sc*AA9A	62%/65%	0.81	-1.50	1.84	90.13%
*Ao*AA9A	53%/57%	0.77	-2.09	2.11	88.10%
*Af*AA9C	50%/55%	0.77	-0.99	2.00	90.50%

*Global Model Quality Estimation. Between 0 and 1, higher values reflect higher reliability.

Is a composite estimator based on different geometrical properties. Scores around 0 indicate that the quality is similar to experimentally determined structure by crystallography. Scores of -4.0 or below are an indication of models with low quality.

Figure S1

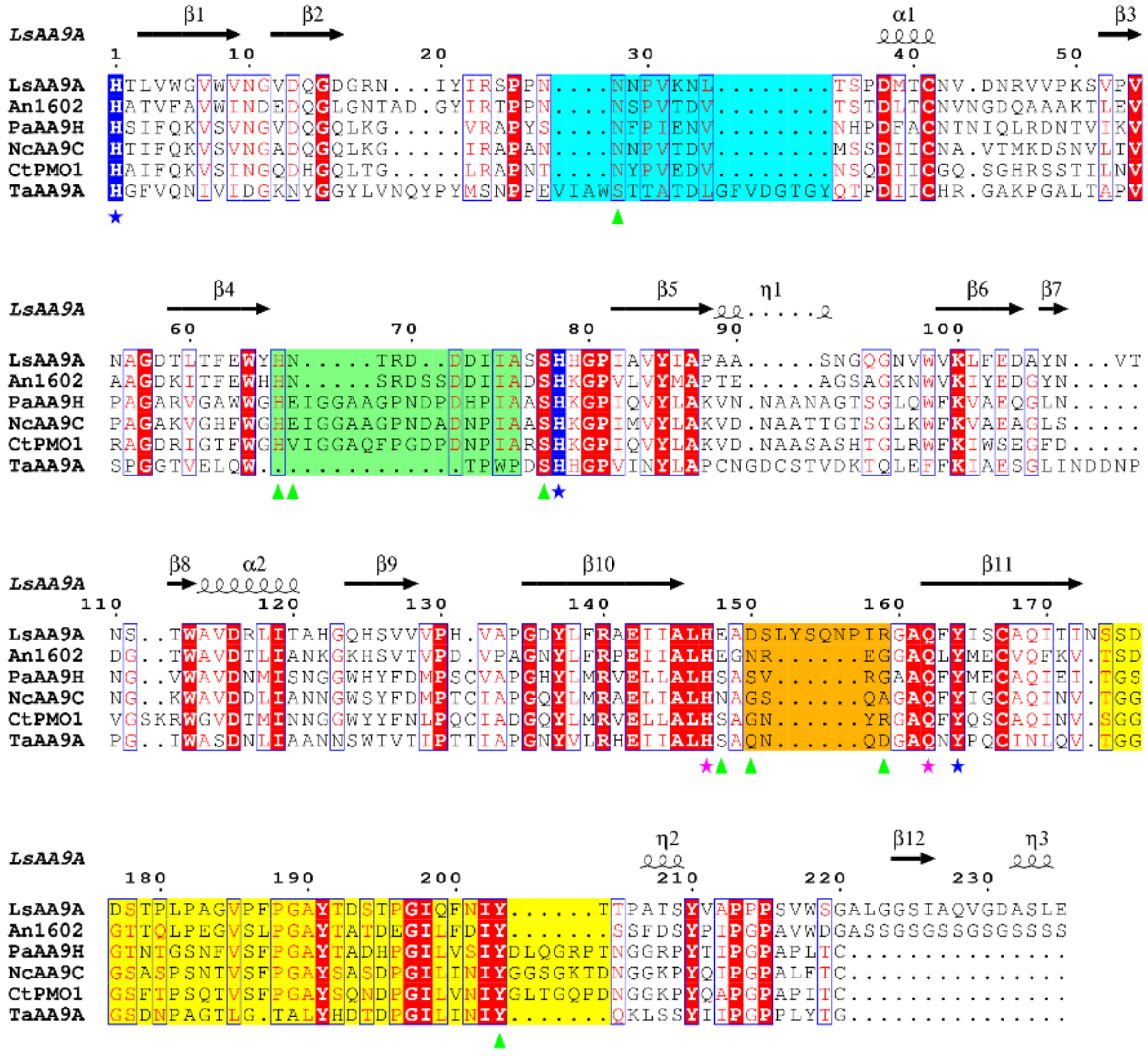

Figure S1. Multiple sequence alignment of AA9 LPMO with previously reported activities on cellooligosaccharide and hemicelluloses. One representative of AA9 LPMOs not active on oligosaccharides, *Ta*AA9A, is included. The His-brace motif is highlighted in blue. Beneath the sequence, positions of the primary or secondary coordination sphere of the copper are highlighted with a blue or a magenta star, respectively and positions involved in cellooligosaccharide interactions with green triangle. The L2, L3, L8 and LC loop regions are colored in cyan, green, orange and yellow, respectively.

Figure S2

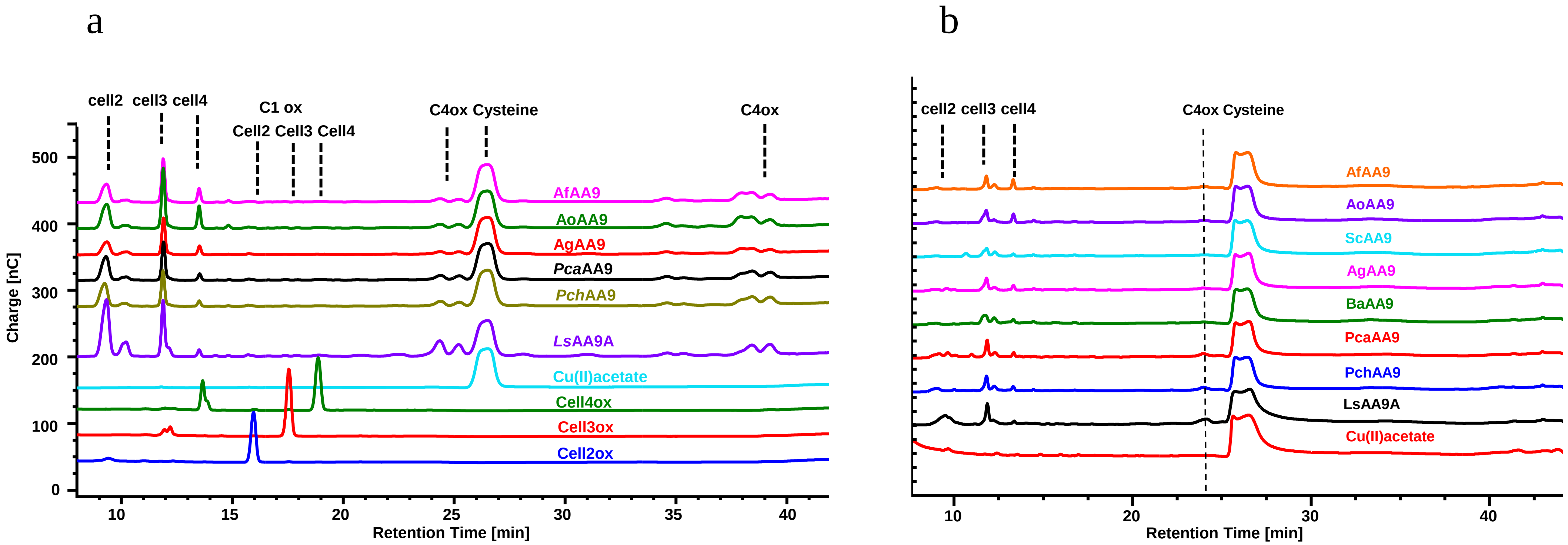

Figure S2. AA9 LPMOs display cellulose activity.
a) Overnight reaction (ca. 20 hours) with AA9 LPMOs and 0.1% (w/v) PASC show the main products formation corresponding to cellobiose (cell2) and cellotriose (cell3). Peaks corresponding to C4 oxidized products are found with retention times of 40 min. No peaks corresponding to C1 oxidized products can be identified. b) Overnight reactions (ca 20 hours) with 0.1% (w/v) Avicel show cell3 as the main product and some evidence of oxidized products. In both cases there are no significant peak formation when replacing the enzyme with equimolar amounts of Cu-acetate.

Figure S3

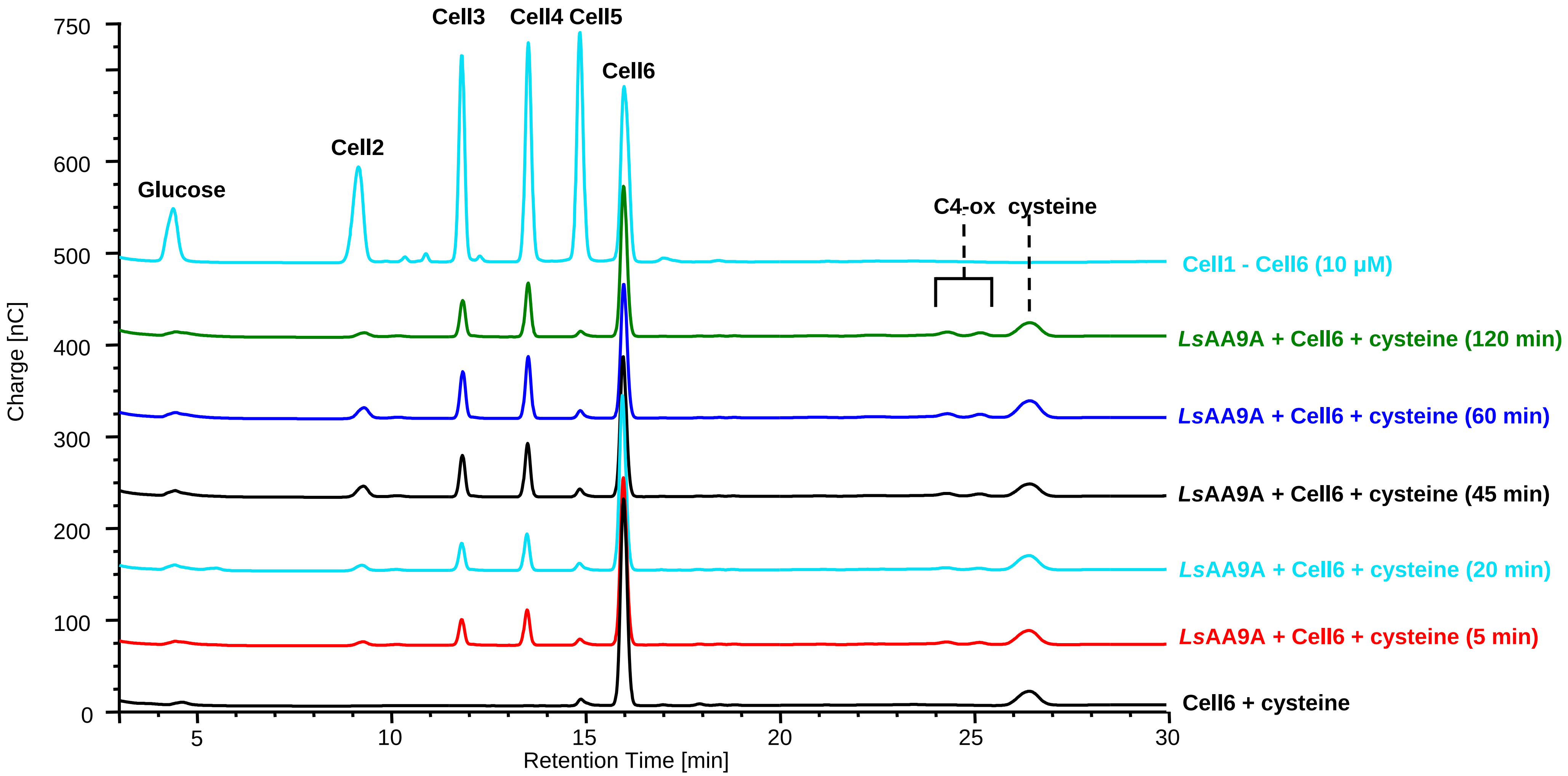

Figure S3. Timecourse degradation of cellohexaose by *Ls*AA9A

From reactions with cell6 the major product formed is cell4 indicating a preferred binding mode from subsite -4 to +2 (as also previously found), but also cell3 products are observed confirming an alternative binding mode from subsite -3 to +3. C4-oxidized products are observed around 25 minutes retention time.

Figure S4

a

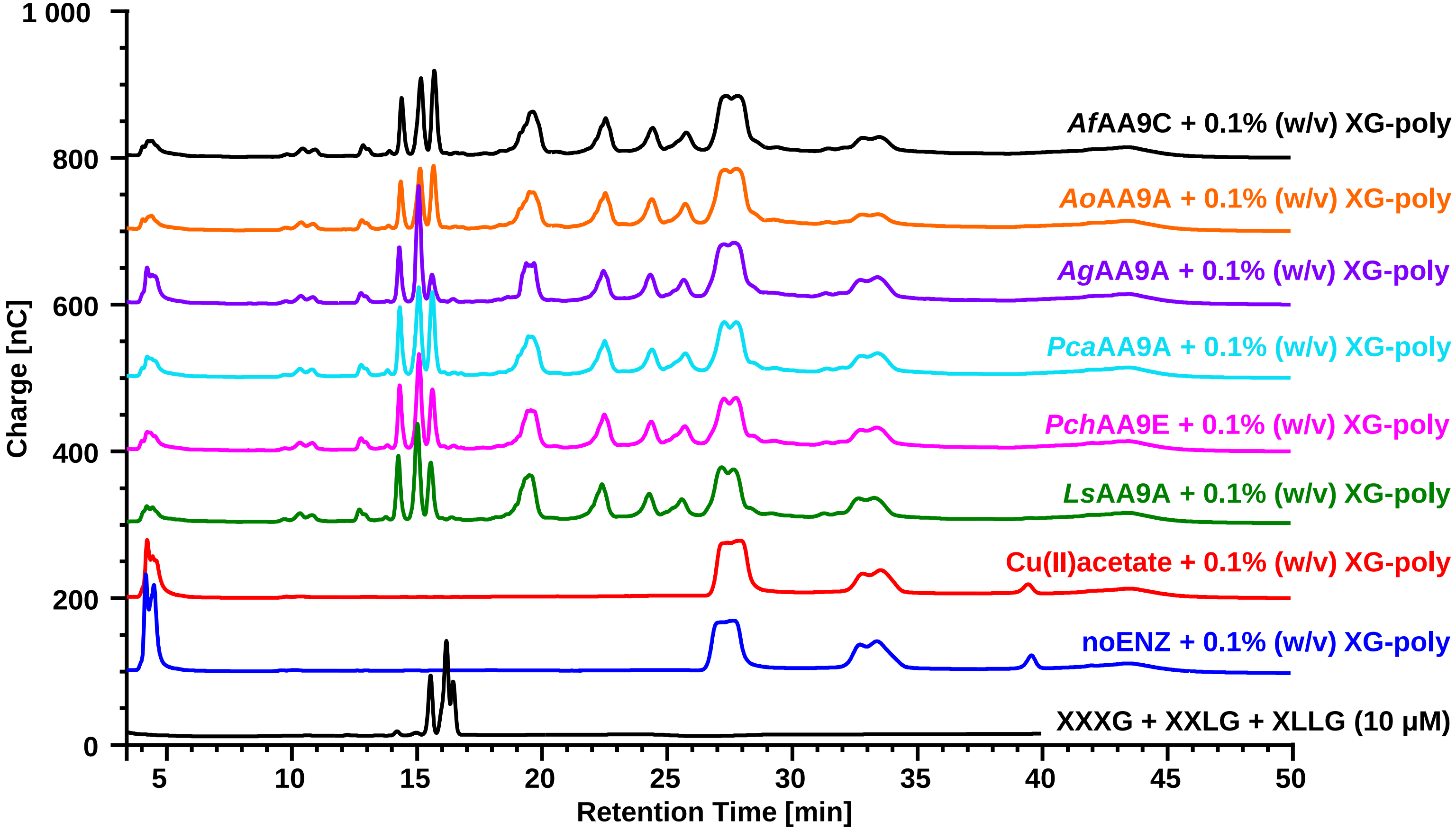

Figure S4. Cleavage of xyloglucan polysaccharide.
Cleavage of xyloglucan polysaccharide after one hour (a) and overnight (b, ca. 22 hours) show a very similar product profile for all AA9 LPMOs. The main products formed during the reaction have retention times similar to a heptasaccharide (*eg* XXXG) or smaller DP.

Figure S4

b

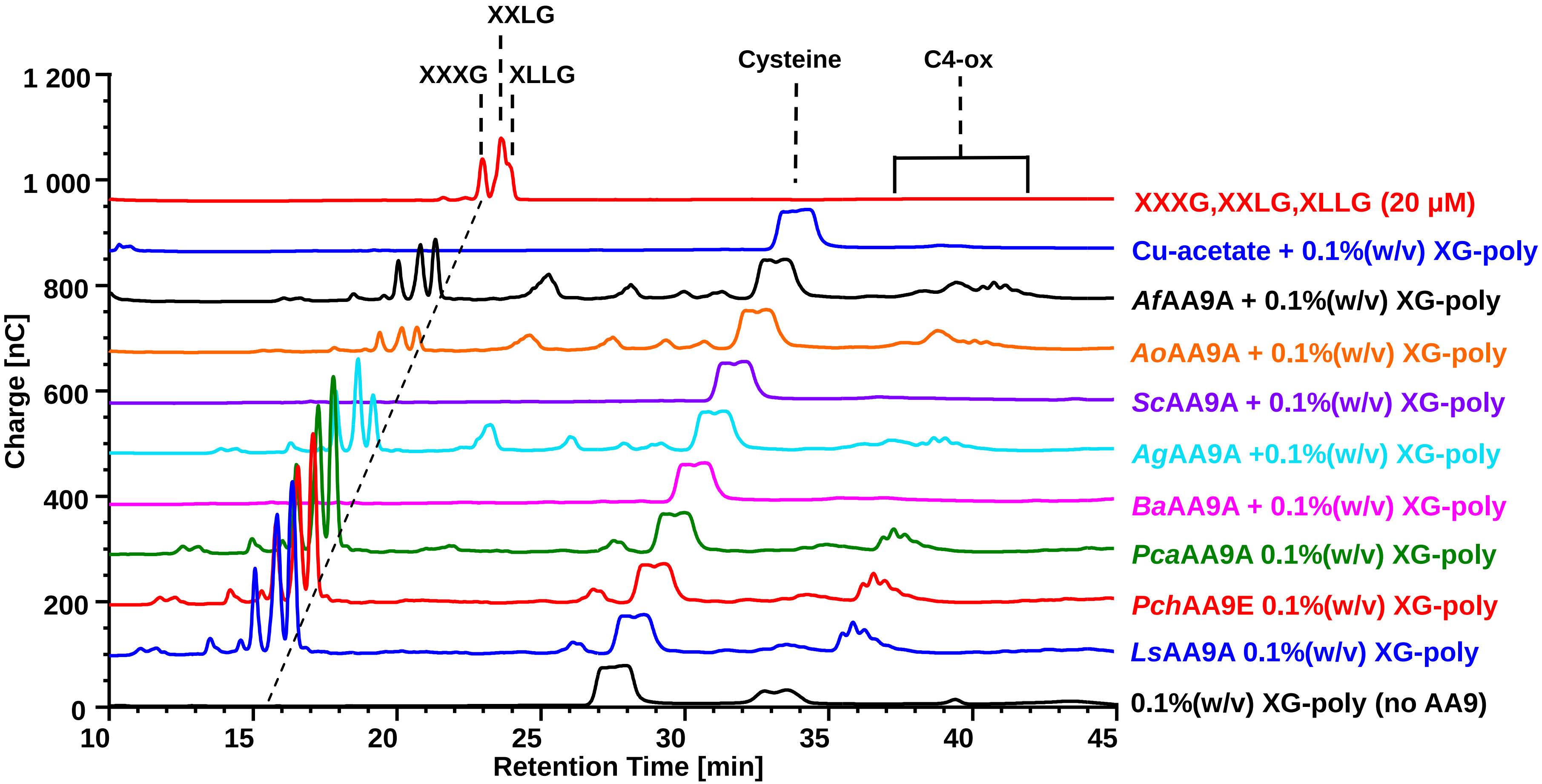

Figure S4. Cleavage of xyloglucan polysaccharide.
Cleavage of xyloglucan polysaccharide after one hour (a) and overnight (b, ca. 22 hours) show a very similar product profile for all AA9 LPMOs. The main products formed during the reaction have retention times similar to a heptasaccharide (*eg* XXXG) or smaller DP.

Figure S5

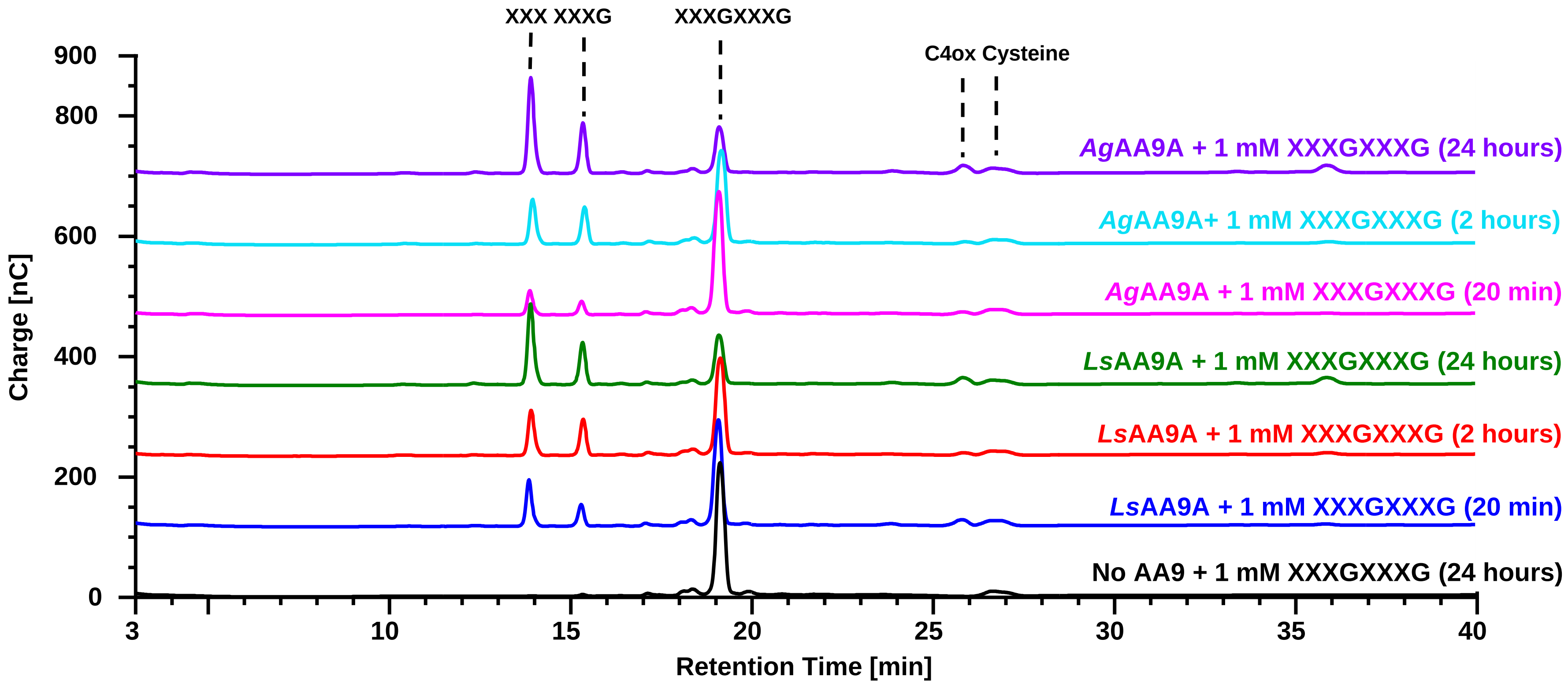

Figure S5. Comparison of *Ls*AA9A and *Ag*AA9A time-course degradation of an XXXGXXXG heptadeca-oligosaccharide.

Both *Ls*AA9A and *Ag*AA9A show a very similar pattern when degrading an XXXGXXXG heptadeca-oligosaccharide, suggesting that the D150Y substitution has little influence on xyloglucan activity. From reactions with XXXGXXXG the major product formed is XXX indicating that both enzymes have a preference for an unsubstituted glucosyl unit in subsite +1.

Figure S6

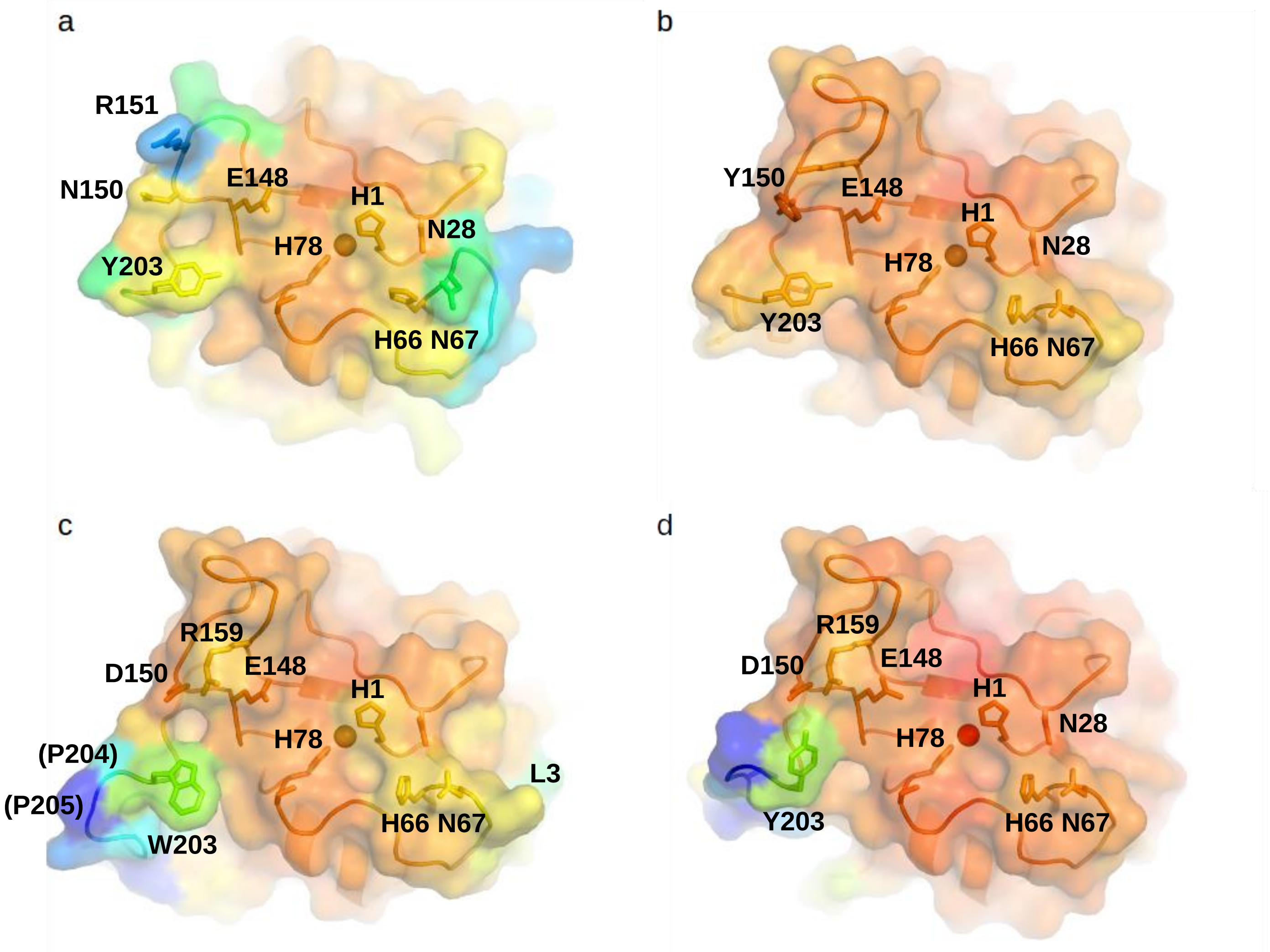

Figure S6. Examples of homology models a) *Ao*AA9A, b) *Ag*AA9A, c) *Sc*AA9A, d) *Pch*AA9E. In the models the QMEAN-Z score is mapped on the structure, with red and blue being the best and worst scores, respectively. Differences in activity may be ascribed to structural difference of the aromatic platform (in the LC loop) near the -3 subsite, although in the models this region is modelled less reliably. Loop regions L2 and L3 seem to be relatively similar.
